# Significant association between three atherosclerosis indexes and stroke risk

**DOI:** 10.1371/journal.pone.0315396

**Published:** 2024-12-19

**Authors:** Xin Wang, Lili Wu, Peng Shu, Wei Yu, Wangfang Yu

**Affiliations:** 1 Department of Neurosurgery, Beilun District People’s Hospital, Ningbo, Zhejiang, China; 2 The Center of Medical Imaging Diagnosis, The First Affiliated Hospital of Wannan Medical College, Wuhu, Anhui; 3 Molecular Laboratory, Beilun District People’s Hospital, Ningbo, Zhejiang, China; Muhimbili University of Health and Allied Sciences School of Medicine, UNITED REPUBLIC OF TANZANIA

## Abstract

**Background:**

To evaluate the associations of three atherosclerosis indexes with stroke in a population aged 65 years and older.

**Methods:**

A sample was obtained from wave 2011 to wave 2015 of the China Health and Retirement Longitudinal Study. Multivariate logistic regression models were used to estimate odds ratios (ORs) with 95% confidence intervals (CIs) for stroke in the quartiles of three atherosclerosis indexes, and restricted cubic splines were constructed.

**Results:**

Four hundred and fifty-four of the 21,913 eligible participants had stroke. After multivariate adjustments and with respect to the lowest quartiles, the ORs (95% CIs) of stroke in the highest quartiles of the atherogenic index of plasma (AIP), the Castelli risk index I (CRI-I), and the Castelli risk index II (CRI-II) were 1.35 (0.99–1.83), 1.52 (1.13–2.06), and 1.40 (1.05–1.86), respectively. When assessed as a continuous exposure, per-unit increases in the AIP, CRI-I, and CRI-II were independently associated with a 49% (OR: 1.49, 95% CI: 1.07–2.08), 6% (OR: 1.06, 95% CI: 1.02–1.11), and 14% (OR: 1.14, 95% CI: 1.03–1.27) increase in the risk of stroke, respectively.

**Conclusion:**

The three atherosclerosis indexes studied—the AIP, CRI-I, and CRI-II—were found to be predictors of stroke in a Chinese population.

## 1. Introduction

Stroke is the second leading cause of death worldwide [1] and thus poses a substantial danger to human health. Over the past 20 years, stroke-related morbidity and mortality have decreased in many developed nations but have increased in low-income and middle-income nations [2]. Moreover, stroke was the leading cause of death in China in recent years [3], and China’s stroke burden was the largest of all countries in the world, as its deaths from stroke constituted almost one third of the total number of deaths from stroke worldwide in 2016 [4]. There are also significant geographical and rural–urban disparities in the incidence and mortality of stroke in China, and stroke incidence and prevalence are both increasing in China [5]. Furthermore, stroke is a significant cause of rapid-onset long-term disability and thus can have serious consequences that lead to decreased physical function and quality of life in patients [6]. Therefore, given the rapidly increasing number of older adults in China, identifying risk factors for stroke may be the most viable approach for reducing the burden of stroke on Chinese society.

Dyslipidemia is one of the most common risk factors for stroke as it leads to atherosclerosis, which is one of main causes of stroke [5, 7]. Dyslipidemia is defined as an increase in the concentrations of low-density lipoprotein cholesterol (LDL-C), total cholesterol (TC), and triglycerides (TGs) and a decrease in the concentration of high-density lipoprotein cholesterol (HDL-C) [8]. Moreover, increased concentrations of TGs and LDL-C and decreased concentrations of HDL-C are associated with an increased incidence of cardiovascular disease (CVD) [9]. Furthermore, some prospective studies have found that increased concentrations of TC may increase total stroke risk and ischemic stroke risk. Furthermore, prospective cohort studies have generally detected an inverse association between HDL-C concentrations and ischemic stroke risk [10, 11].

In recent years, researchers have recognized that abnormal plasma lipid profiles are key risk factors for and predictors of CVD. Accordingly, individual lipid variables, such as concentrations of TC, LDL, and HDL-C, were suggested to be valuable for use in the prediction of CVD risk [12]. Consequently, various tools have been developed as potentially meaningful and practical biomarkers to predict atherosclerosis and CVD. For example, Castelli risk indexes I and II (CRI-I and CRI-II), the atherogenic index of plasma (AIP), and the atherogenic coefficient (AC), known as atherosclerosis indexes, are biomarkers for evaluating CVD risk. The AIP is the logarithmically transformed ratio of TG concentration to HDL-C concentration [13]; CRI-I and CRI-II are calculated as TC concentration/HDL-C concentration and LDL-C concentration/HDL-C concentration, respectively; and AC is calculated as TC concentration − HDL-C concentration/HDL-C concentration [14]. Thus, the abovementioned atherosclerosis indexes are comprehensive indicators based on parameters that are inexpensive and simple to measure.

However, there has been little research on the associations of the AIP, CRI-I, and CRI-II, as CVD indicators, with stroke in Chinese populations. Therefore, the purpose of this study was to identify the associations between the aforementioned atherosclerosis indexes and stroke in a population aged 65 years and older from the China Health and Retirement Longitudinal Study (CHARLS).

## 2. Methods

### 2.1 Study population

The CHARLS, which started in 2011 and has follow-ups every 2 years, is a longitudinal study that gathers information on the social, economic, and general health status of community-dwelling people from 28 provinces in mainland China. All of the participants provided informed consent, and the protocol was approved by the Institutional Review Board at Peking University. We used data from the CHARLS survey’s 2011 and 2015 waves in the current study. We used aged 65 years and older as our inclusion criterion, which yielded 21,913 participants. Further information can be found elsewhere [15].

### 2.2 Atherosclerosis indexes

We assessed three atherosclerosis indexes at baseline, namely, the AIP, CRI-I, and CRI-II, and calculated them using the following formulas.

1. AIP = lg (TG concentration/HDL-C concentration)
2. CRI-I = TC concentration/HDL-C concentration
3. CRI-II = LDL-C concentration/HDL-C concentration

Subsequently, we categorized the participants into quartiles based on their calculated atherosclerosis indexes.

### 2.3 Stroke

Self-reported stroke was determined using the following question: “Have you ever been diagnosed with stroke by a doctor?” The participants who answered “Yes” were regarded as having had a stroke, whereas the participants who answered “No” were regarded as not having had a stroke [16].

### 2.4 Covariates

The sociodemographic characteristics considered were age, sex (female/male), marital status (non-married/married), and living location (urban/rural). The lifestyle variables considered were smoking and alcohol drinking. The health status and clinical measures considered were use of antihypertensive drugs, use of hypolipidemic drugs, use of lipid-lowering drugs, presence of hypertension, presence of dyslipidemia, presence of diabetes, and presence of increased concentrations of C-reactive protein (CRP). Hypertension was defined as a mean systolic blood pressure ≥ 140 mmHg and/or a mean diastolic blood pressure ≥ 90 mmHg, or any self-reported history of physician-diagnosed hypertension. Dyslipidemia was defined as a TC concentration > 6.2 mmol/L, a TG concentration ≥ 2.3 mmol/L, an LDL-C concentration ≥ 4.1 mmol/L, an HDL-C concentration < 1.0 mmol/L in men or < 1.3 mmol/L in women, or having any self-reported history of physician-diagnosed dyslipidemia. Diabetes was defined as a fasting plasma glucose concentration ≥ 7.0 mmol/L and/or a glycated hemoglobin (HbA1c) concentration ≥ 6.2 mmol/L, or any self-reported history of physician-diagnosed diabetes [17].

### 2.5 Statistical analyses

Continuous variables are presented as means with standard deviations and were compared using rank-sum tests and t-tests, whereas categorical variables are presented as counts and proportions and were compared using chi-square tests. Multivariate logistic regressions were performed to estimate the odds ratios (ORs) and 95% confidence intervals (CIs) for stroke. The adjusted model included four models. Model 1 was a rough model with no adjustment. Model 2 was adjusted for age, body mass index (BMI), sex, living location, and marital status. Model 3 was additionally adjusted for smoking status and alcohol-drinking status, antihypertensive drug use, hypolipidemic drug use. Model 4 was additionally adjusted for the presence of hypertension, dyslipidemia, diabetes, and CRP.

Moreover, restricted cubic splines of the atherosclerosis indexes were plotted as continuous variables to examine these indexes’ associations with the risk of stroke. Stratified analyses were conducted to assess the potential modifying effects of the following variables: age (= 60 vs. ≥ 60 years), sex (male vs. female), marital status (non-married vs. married), living location (urban vs. rural), smoking status (smoker vs. non-smoker), alcohol-drinking status (alcohol drinker vs. non-alcohol drinker), current hypertension (“yes” vs. “no”), current dyslipidemia (“yes” vs. “no”), and current diabetes (“yes” vs. “no”).

All of the analyses were performed using SAS (Version 9.4, SAS Institute, Cary, North Carolina). Two-sided p-values of ≤ 0.05 were regarded as indicating statistical significance.

## 3. Results

The characteristics of the participants are presented in Table 1. Four hundred and fifty-four of the 21,913 participants had stroke. Compared with the participants without stroke, those with stroke were older (59.54 ± 9.93 years old vs. 64.48 ± 9.37 years old, p < 0.001); had a higher BMI (23.74 ± 3.74 kg/m2 vs. 24.48 ± 3.90 kg/m2, p < 0.001); were more likely to be men (45.9% vs. 51.3%, p = 0.02) and smokers (40.8% vs. 47.6%, p = 0.004); and were less likely to be alcohol drinkers (34.1% vs. 24.7%, p < 0.001). In addition, compared with the participants without stroke, those with stroke had significantly higher concentrations of TC (p = 0.003), TGs (p = 0.001), HDL-C (p < 0.001), non-HDL-C (p = 0.002), fasting plasma glucose (p < 0.001), and HbA1c (p < 0.001).

**Table 1:** Baseline characteristics of participants with/without stroke (N = 21,913).

	Total	Non-stroke	stroke	*P*
	(N = 21913)	(N = 21459)	(N = 454)	
Age, year	59.64±9.95	59.54±9.93	64.48±9.37	<0.001
Gender				0.02
Female	11832 (54.0)	11611 (54.1)	221 (48.7)	
Male	10081 (46.0)	9848 (45.9)	233 (51.3)	
BMI, kg/m2	23.75±3.74	23.74±3.74	24.48±3.90	<0.001
Marita				0.04
Non-Married	2760 (12.6)	2688 (12.5)	72 (15.9)	
Married	19153 (87.4)	18771 (87.5)	382 (84.1)	
Location				0.003
Urban	17376 (79.3)	17042 (79.4)	334 (73.6)	
Rural	4537 (20.7)	4417 (20.6)	120 (26.4)	
Smoking	8977 (41.0)	8761 (40.8)	216 (47.6)	0.004
Drinking	7427 (33.9)	7315 (34.1)	112 (24.7)	<0.001
Antihypertensive drugs	4893 (22.3)	4651 (21.7)	242 (53.3)	<0.001
Hypolipidemic drugs	1043 (4.8)	988 (4.6)	55 (12.1)	<0.001
Lipid-lowering drugs	1426 (6.5)	1333 (6.2)	93 (20.5)	<0.001
Hypertension	5097 (23.3)	4826 (22.5)	271 (59.7)	<0.001
Dyslipidemia	2074 (9.5)	1934 (9.0)	140 (30.8)	<0.001
Diabetes	1218 (5.6)	1155 (5.4)	63 (13.9)	<0.001
WC, cm	6.10±1.98	6.10±1.98	6.37±2.05	0.003
TC, mg/dl	187.88±37.51	187.88±37.52	187.84±37.16	0.98
TG, mg/dl	137.59±92.48	137.29±92.35	151.74±97.59	0.001
HDL_C, mg/dl	51.22±13.25	51.30±13.23	47.13±13.51	<0.001
LDL_C, mg/dl	108.27±32.41	108.26±32.41	109.00±32.64	0.62
Non-HDLC, mg/dl	113.07±80.37	112.82±80.33	124.71±81.20	0.002
Glucose, mg/dl	106.25±35.66	106.11±35.61	112.78±37.17	<0.001
HbA1c, %	5.67±0.98	5.67±0.98	5.87±1.18	<0.001
CRP, mg/l	2.75±6.83	2.73±6.85	3.47±5.98	0.02
AIP	0.37+0.30	0.37+0.30	0.45+0.31	<0.001
CRI-I	3.88+1.35	3.87+1.35	4.24+1.37	<0.001
CRI-II	2.22+0.82	2.22+0.82	2.44+0.89	<0.001

Chi-square test, rank-sum test and t-test were used to calculate p-values for categorical variables, continuous variables without normal distribution, and continuous variables with normal distribution, respectively.

WC, waist circumference; TC, total cholesterol; TG, triglyceride; HDL_C, high-density lipoprotein cholesterol; LDL_C, low-density lipoprotein cholesterol; Non-HDLC, TC-HDL_C; CRP, C-reaction protein; HbA1C, glycated haemoglobin; AIP, atherogenic Index of Plasma; CRI-I, Castelli’s risk index-I; CRI-II, Castelli’s risk index

### 3.1 Associations of the AIP, CRI-I, and CRI-II with stroke

**Fig 1:**
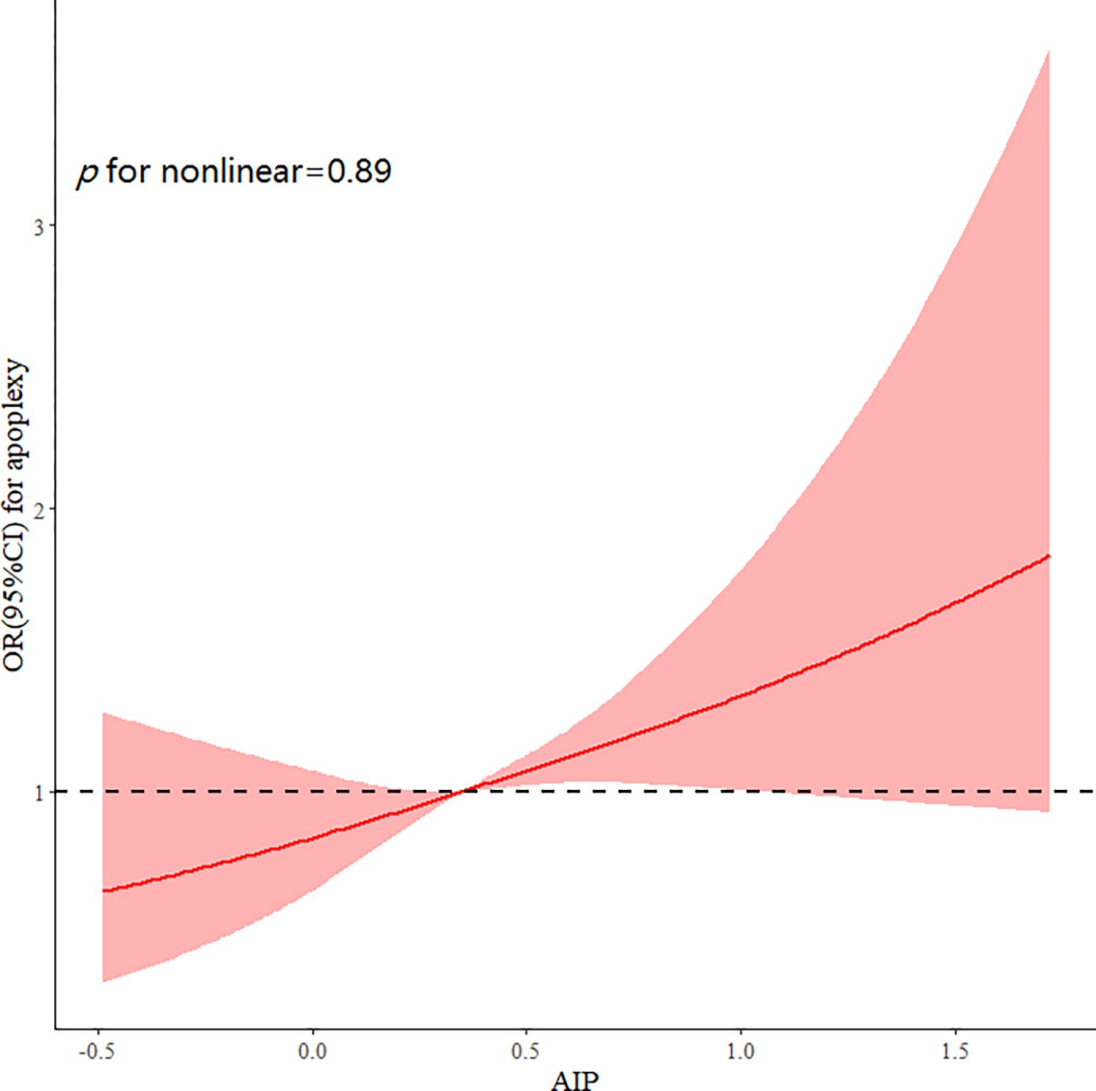
Adjusted cubic spline models of the association between AIP and risk of incident stroke.

There was a linear association between the AIP and stroke (p = 0.89 for nonlinearity) (Fig 1), and high AIPs were associated with a high risk of stroke. The multivariable ORs of stroke were 1.10 (95% CI: 0.81–1.50) in the second quartile, 1.40 (95% CI: 1.04–1.89) in the third quartile, and 1.35 (95% CI: 0.99–1.83) in the fourth quartile compared with the reference group (the first quartile) (Table 2).

**Table 2:** Associations between the atherosclerosis indexes and incident stroke in the CHARLS.

Atherosclerosis indexes		Quartile 1	Quartile 2	Quartile 3	Quartile 4	per unit increase	P for trend
Atherogenic Index of Plasma	Case/N	76/5479	94/5478	134/5478	150/5478		
	Model1	Reference	1.24 (0.92–1.68)	1.78 (1.34–2.37)	2 (1.51–2.64)	2.29 (1.72–3.06)	<0.001
	Model2	Reference	1.18 (0.87–1.61)	1.61 (1.2–2.16)	1.78 (1.33–2.4)	2.09 (1.52–2.86)	<0.001
	Model3	Reference	1.1 (0.81–1.5)	1.42 (1.05–1.91)	1.42 (1.05–1.92)	1.6 (1.15–2.22)	0.009
	Model4	Reference	1.1 (0.81–1.5)	1.4 (1.04–1.89)	1.35 (0.99–1.83)	1.49 (1.07–2.08)	0.025
Castelli’s risk index-I	Case/N	72/5479	106/5478	111/5478	165/5478		
	Model1	Reference	1.48 (1.1–2)	1.55 (1.15–2.09)	2.33 (1.76–3.08)	1.1 (1.05–1.16)	<0.001
	Model2	Reference	1.38 (1.02–1.87)	1.35 (0.99–1.84)	1.95 (1.46–2.62)	1.08 (1.03–1.13)	<0.001
	Model3	Reference	1.3 (0.96–1.77)	1.23 (0.9–1.67)	1.64 (1.21–2.21)	1.07 (1.03–1.11)	0.002
	Model4	Reference	1.31 (0.97–1.79)	1.22 (0.89–1.66)	1.52 (1.13–2.06)	1.06 (1.02–1.11)	0.014
Castelli’s risk index-II	Case/N	80/5479	101/5478	116/5478	157/5478		
	Model1	Reference	1.27 (0.94–1.7)	1.46 (1.1–1.95)	1.99 (1.52–2.61)	1.3 (1.19–1.43)	<0.001
	Model2	Reference	1.17 (0.87–1.57)	1.26 (0.94–1.69)	1.64 (1.24–2.17)	1.23 (1.12–1.36)	<0.001
	Model3	Reference	1.13 (0.83–1.52)	1.19 (0.89–1.6)	1.5 (1.13–2)	1.18 (1.07–1.31)	0.003
	Model4	Reference	1.12 (0.83–1.52)	1.17 (0.87–1.57)	1.4 (1.05–1.86)	1.14 (1.03–1.27)	0.018

Model 1 was rough model; Model 2 adjusted for age, BMI, gender, location and marriage; Model 3 further adjusted for smoking, drinking, Antihypertensive drugs, Hypolipidemic drugs, Lipid-lowering drugs; Model4 further adjusted hypertension, dyslipidemia, diabetes, and CRP; AI, Index of atherogenicity.

**Fig 2:**
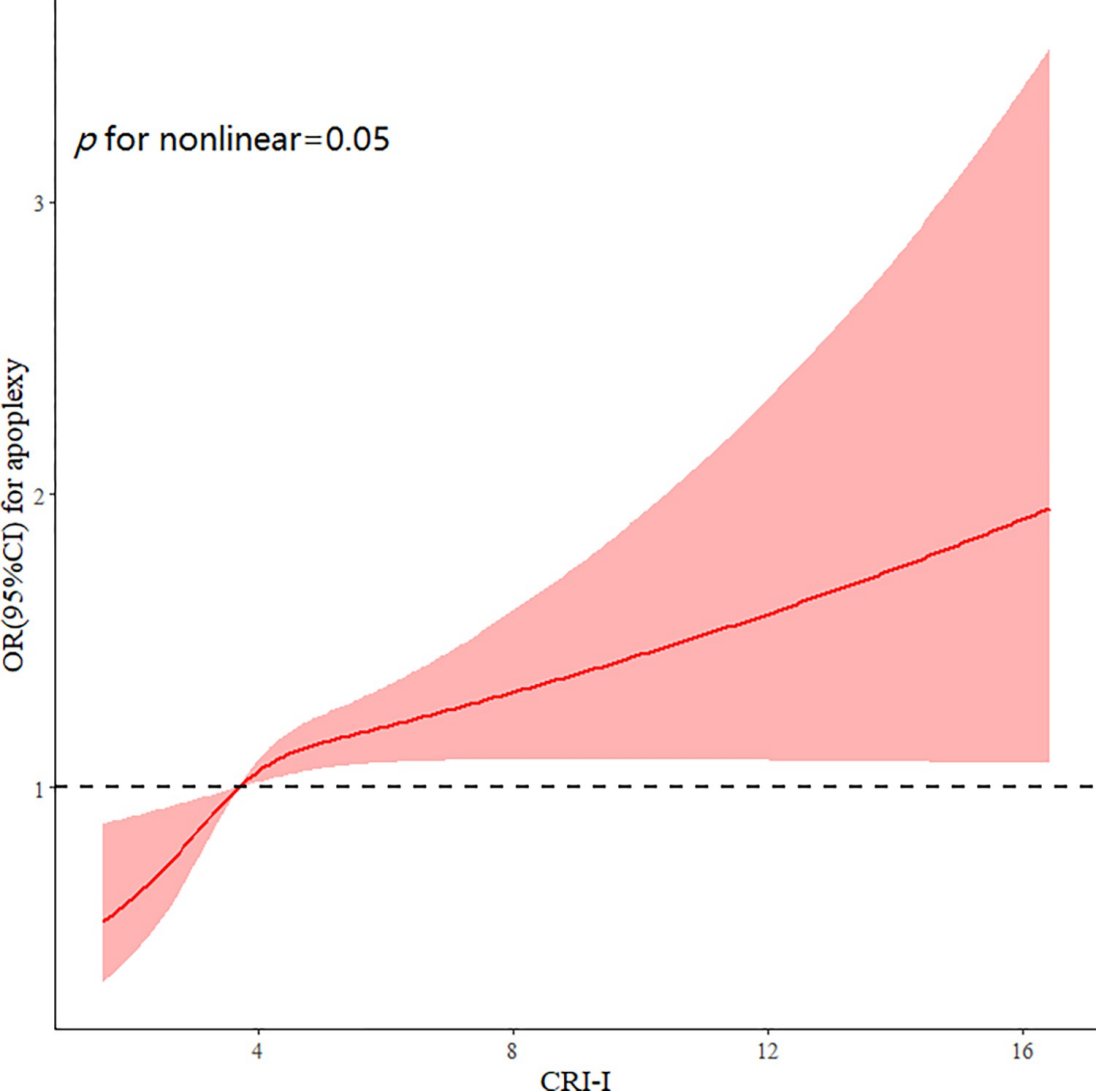
Adjusted cubic spline models of the association between CRI-I and risk of incident stroke.

There was a nonlinear association between the CRI-I and stroke (p = 0.05 for nonlinearity) (Fig 2). Compared with the reference group (the first quartile), the multivariable-adjusted model ORs of stroke were significantly increased in the other quartiles, that is, 1.31 (95% CI: 0.97–1.79) in the second quartile, 1.22 (95% CI: 0.89–1.66) in the third quartile, and 1.52 (95% CI: 1.13–2.06) in the fourth quartile (Table 2).

**Fig 3:**
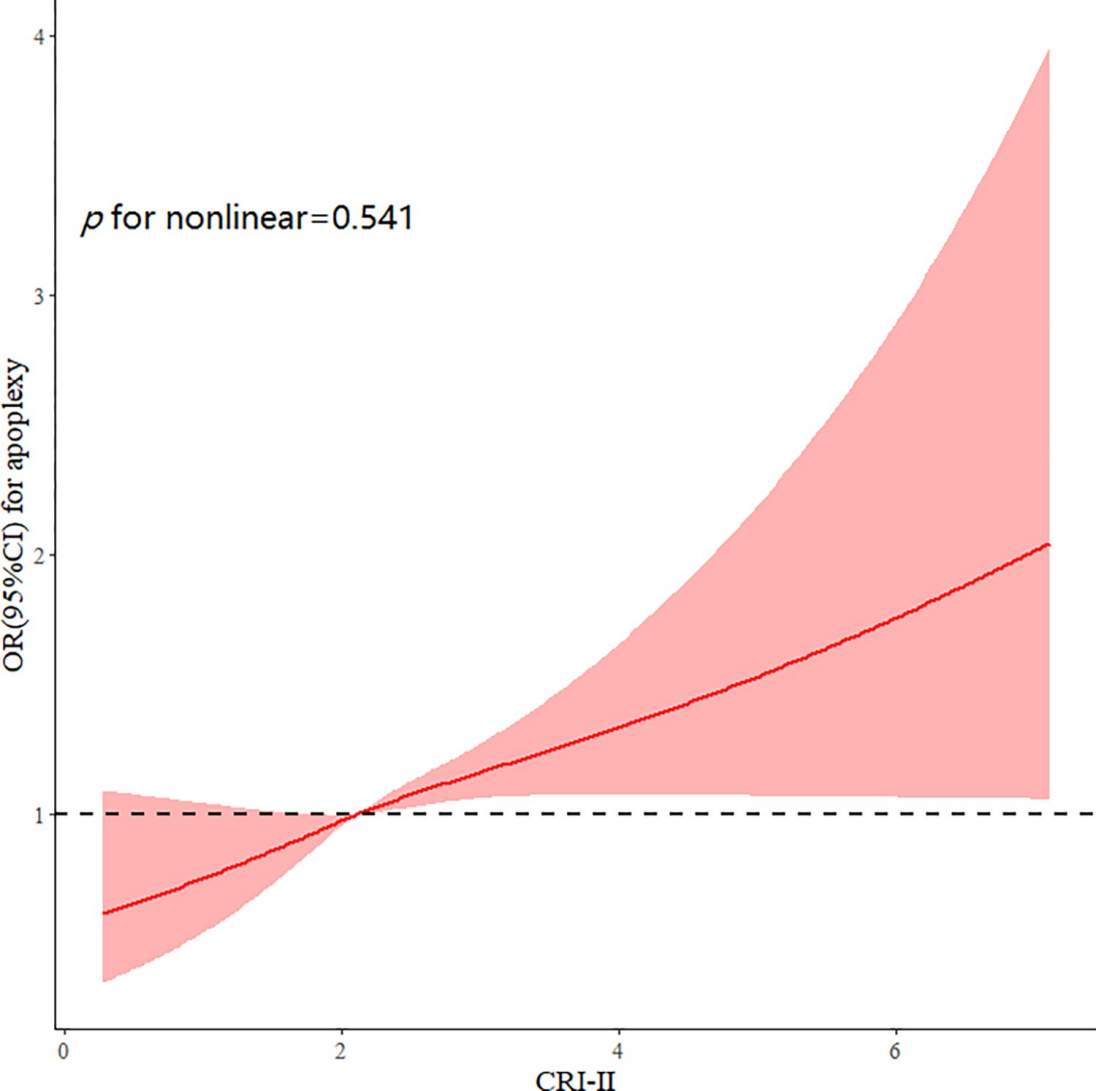
Adjusted cubic spline models of the association between CRI-II and risk of incident stroke.

There was a linear association between the CRI-II and stroke (p = 0.541 for nonlinearity) (Fig 3), and high CRI-II values were associated with a high risk of stroke. Compared with the reference group (the first quartile), the multivariate-adjusted model ORs of stroke were increased in the other quartiles, that is, 1.12 (95% CI: 0.83–1.52) in the second quartile, 1.17 (95% CI: 0.87–1.57) in the third quartile, and 1.40 (95% CI: 1.05–1.86) in the fourth quartile (Table 2).

In addition, when assessed as continuous exposures, per-unit increases in AIP, CRI-I, and CRI-II values were independently associated with a 49% (OR: 1.49, 95% CI: 1.07–2.08), 6% (OR: 1.069, 95% CI: 1.02–1.11), and 14% (OR: 1.14, 95% CI: 1.03–1.27) decrease in the risk of stroke, respectively (Table 2).

### 3.2 Subgroup analyses

**Fig 4:**
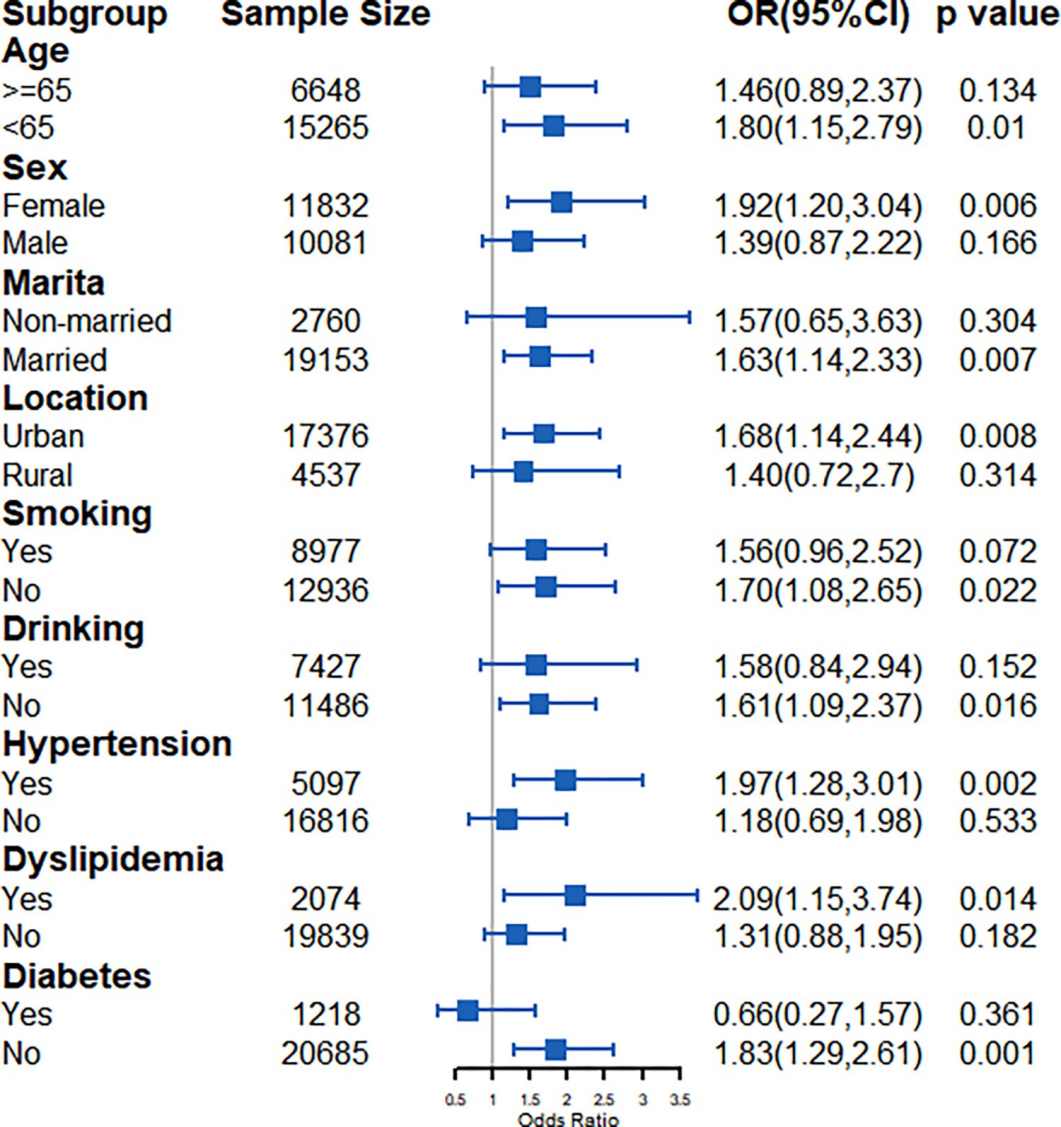
Subgroup analysis for AIP.

In the AIP subgroup, a high AIP was significantly associated with a high risk of stroke in the participants who were aged under 65 years (OR: 1.80, 95% CI: 1.15–2.79), female (OR: 1.92, 95% CI: 1.20–3.04), married (OR: 1.63, 95% CI: 1.14–2.33), living in an urban area (OR: 1.68, 95% CI: 1.14–2.44), or non-smokers (OR: 1.70, 95% CI: 1.08–2.65) and alcohol drinkers (OR: 1.61, 95% CI: 1.09–2.37) and had hypertension (OR: 1.97, 95% CI: 1.28–3.01) and dyslipidemia (OR: 2.09, 95% CI: 1.15–3.74) or did not have diabetes (OR: 1.83, 95% CI: 1.29–2.61) (Fig 4).

**Fig 5:**
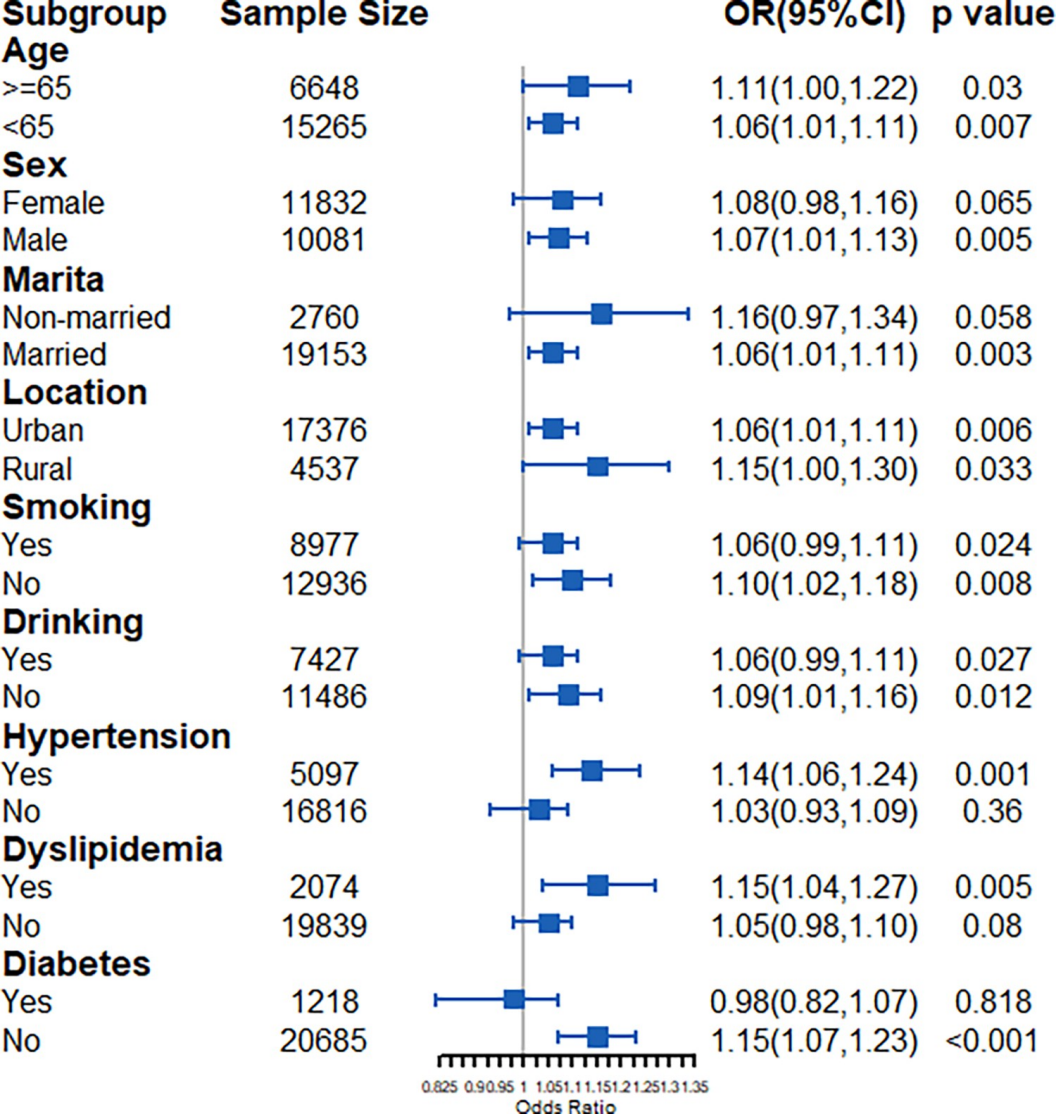
Subgroup analysis for CRI-I.

In the CRI-I subgroup, the association between the CRI-I and stroke was generally consistent in the participants who were men (OR: 1.07, 95% CI: 1.01–1.13), married (OR: 1.06, 95% CI: 1.01–1.11), non-smokers (OR: 1.10, 95% CI: 1.02–1.18), and alcohol drinkers (OR: 1.09, 95% CI: 1.01–1.16) and had hypertension (OR: 1.14, 95% CI: 1.06–1.24) and dyslipidemia (OR: 1.15, 95% CI: 1.04–1.27) or did not have diabetes (OR: 1.15, 95% CI: 1.07–1.23) (Fig 5).

**Fig 6:**
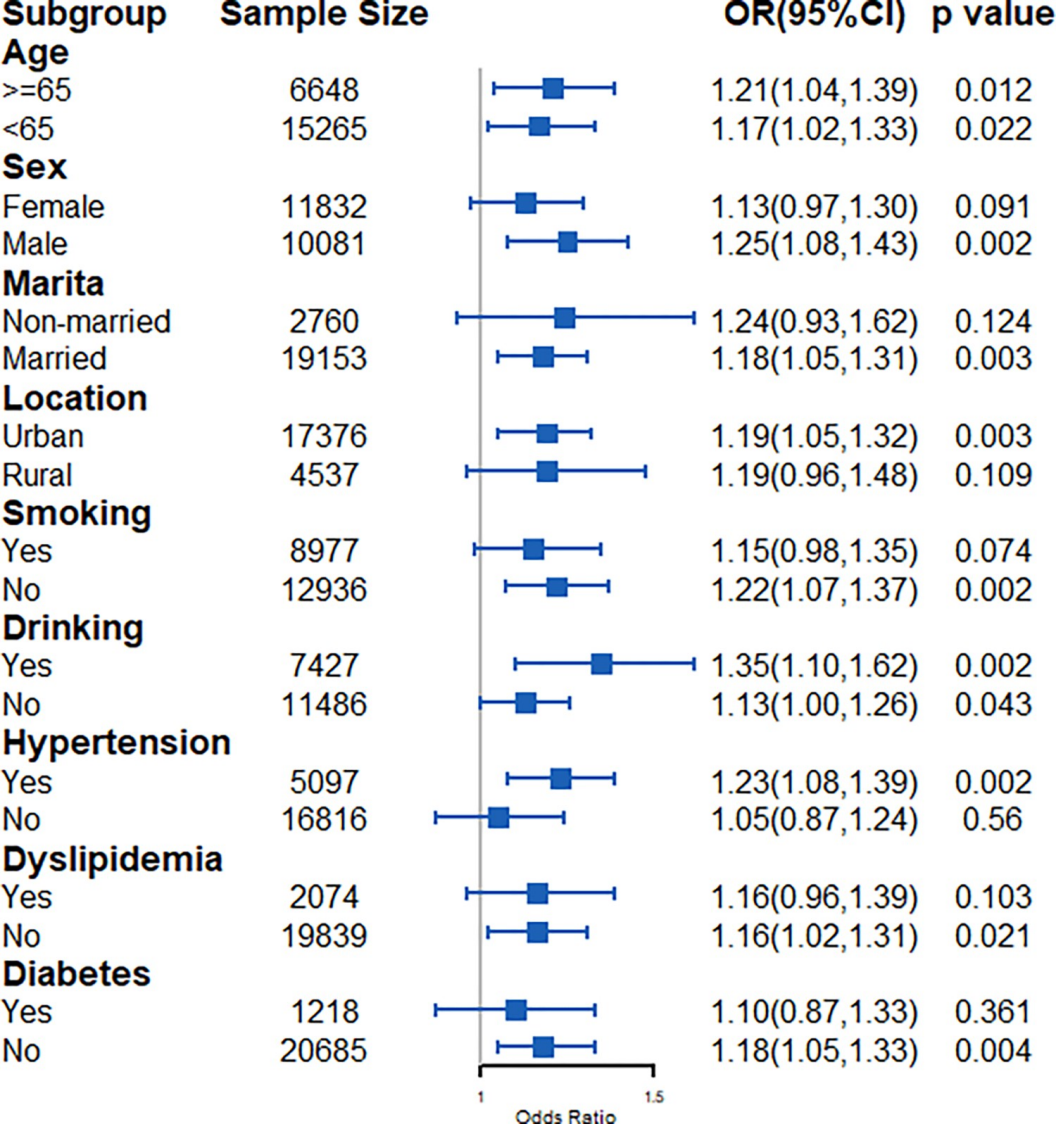
Subgroup analysis for CRI-II.

In the CRI-II subgroup, a high CRI-II was associated with a high risk of stroke in the participants who were male (OR: 1.25, 95% CI: 1.08–1.43), married (OR: 1.18, 95% CI: 1.05–1.31), and non-smokers (OR: 1.22, 95% CI: 1.07–1.37) and who had hypertension (OR: 1.23, 95% CI: 1.08–1.39) and dyslipidemia (OR: 1.16, 95% CI: 1.02–1.31), or did not have diabetes (OR: 1.05, 95% CI: 1.05–1.33) (Fig 6).

## 4. Discussion

We analyzed the baseline and follow-up data of 21,913 eligible participants in a prospective cohort from the CHARLS to explore the associations between three atherosclerosis indexes and stroke risk. The results indicate that high AIPs and high CRI-I and CRI-II values were significantly correlated with an increased risk of stroke, and a cubic spline model suggested that there was a significant nonlinear dose–response relationship between the CRI-I and stroke risk. Furthermore, subgroup analyses suggested that variations in the characteristics (i.e., sex, lifestyle, and comorbidity characteristics) of the study population might have affected the association between the atherosclerosis indexes and stroke risk.

Atherosclerosis is thickening of the intima of arteries caused by plaques formed by accumulation of lipids and/or fibrous substances and is a key biological precursor state to ischemic heart disease and ischemic stroke [18]. Indexes used in clinical lipid analysis, such as the CRI-I (TC concentration/HDL-C concentration) and CRI-II (LDL-C concentration/HDL-C concentration), are considered reliable and comprehensive indicators of lipid metabolism disorders [19]. The CRI-I reveals the presence of coronary artery plaques, and the CRI-II has been proven to be an excellent predictor of cardiovascular risk. Compared with the CRI-I, the CRI-II is more predictive of insulin resistance [20] and acute myocardial infarction [21]. Furthermore, the AIP is a new blood lipid index devised by Dobiásová et al. [22]. Compared with the CRI-I and CRI-II, the AIP more comprehensively reflects the impairment of plasma lipoprotein metabolism and the presence of inflammation in vivo and better reflects the extent of atherosclerosis [23]. Moreover, recent studies have found that the AIP better predicts the risk of diabetes, metabolic syndrome, and hypertension in middle-aged people than the conventional method, i.e., lipid mass spectrometry [24, 25].

Furthermore, Liu et al. [25] prospectively recruited 1463 patients with acute ischemic stroke and investigated the relationship between the AIP and adverse outcomes of acute ischemic stroke. Their results showed that a high AIP was associated with adverse outcomes in these patients (OR: 1.84, 95% CI: 1.23–2.53). Wang et al. [26] evaluated the ability of the AIP to predict the risk of ischemic stroke in 5428 residents in rural areas of Northeast China. Their analysis showed that the AIP significantly enhanced the ability to estimate ischemic stroke in women and men (net reclassification improvement = 0.188 and 0.175, respectively). A recent study based on the CHARLS evaluated the performance of a baseline arteriosclerosis index in predicting type 2 diabetes mellitus (T2DM). The results showed that a high AIP and a high CRI-I value were associated with a high risk of T2DM [ORs (95% CI) of 1.29 (1.18–1.42) and 1.41 (1.25–1.59), respectively] [27]. The current study was also based on the CHARLS and is the first exploration of the ability of the AIP, CRI-I, and CRI-II to predict stroke risk. Our results suggest that the aforementioned atherosclerosis indexes are effective independent predictors of stroke risk.

The AIP, CRI-I, CRI-II, and other atherosclerosis indexes are calculated using multiple blood lipid parameters typically measured via lipid mass spectrometry, and thus, these indexes’ clinical significance should be greater than that of indexes calculated using a single lipid parameter. Increases in TC and/or TG concentrations are the main clinical manifestation of dyslipidemia, whereas HDL-C is a protective factor for cardiovascular disease. As a lipid concentration is a continuous variable, there is no natural cut-off point between normal and abnormal concentrations. However, an increase in various atherosclerosis indexes tends to reflect increases in the plasma concentrations of TGs and LDL-C and decreases in the plasma concentration of HDL-C [28]. Therefore, a high atherosclerosis index value is a reliable indicator of the presence of dyslipidemia, which is an important risk factor for stroke [29]. Epidemiological data show that dyslipidemia can increase the risk of ischemic cerebrovascular accident [30]. In addition, there is increasing evidence that insulin resistance related to metabolic syndrome is the main risk factor for atherosclerotic cardiovascular disease, cerebrovascular accidents, and peripheral arterial disease, the global mortality rates of which have increased by nearly 1.5 times compared with 1990 [31].

Our results suggest that early interventions to treat dyslipidemia may reduce the risk of stroke in populations and help to alleviate the related cardiovascular disease burden. For example, lifestyle changes can help to normalize TC, TG, LDL-C, and HDL-C concentrations [31]. Therefore, patients with dyslipidemia should be informed of the importance of changing their behavior and lifestyle, regardless of whether they are prescribed anti-dyslipidemic medications. However, some lifestyle changes, such as diet and physical activity, are unlikely to significantly reduce blood lipid concentrations, and thus, many patients with hyperlipidemia also require medication to achieve treatment goals [32].

In the current study, subgroup analyses revealed that the associations between the three atherosclerosis indexes and stroke risk may differ between populations and depend on their respective characteristics. Specifically, we found that the ability of the CRI-I and CRI-II to predict stroke was significant only in men, whereas the ability of the AIP to predict stroke was significant only in women. Due to sex-related differences in body fat distribution and hormone regulation, there is significant heterogeneity in lipid disorders between males and females [33]. Estrogen is a vascular protective agent and thus can accelerate the clearance of TC from the body, improve endothelial function, and reduce deposition of blood lipids [34]. The beginning of menopause leads to decreases in estrogen concentrations, which may result in menopausal metabolic syndrome (insulin resistance, abdominal obesity, and dyslipidemia) [35].

In addition, sex-affected lifestyle factors, including smoking and alcohol drinking, also affect the association between atherosclerosis indexes and stroke risk. In the current study, we found that the predictive power of the atherosclerosis indexes was generally greater in non-smokers and non-alcohol drinkers than in smokers and alcohol drinkers, aside from the CRI-II having greater predictive power in alcohol drinkers than in non-alcohol drinkers (OR: 1.35 vs. 1.13). Smoking and excessive alcohol drinking are independent risk factors for cardiovascular disease [36, 37]. Moreover, hypertension and dyslipidemia are the most important cardiovascular risk factors in the primary prevention of ischemic heart disease [38]. The stronger correlations that we observed between the atherosclerosis indexes and stroke risk in hypertensive and dyslipidemic populations than in non-hypertensive and non-dyslipidemic populations suggest that joint interventions for hypertension and dyslipidemia should be implemented. However, we found that the abovementioned correlation was weaker in a diabetes subpopulation. This is consistent with the results of Wu et al. [27], which showed that atherosclerosis indexes have differing abilities to predict the occurrence of T2DM. Thus, further research is needed to determine the mechanisms underlying such indexes’ predictive abilities.

The three atherosclerosis indexes are cost-effective and convenient and can be used, and their results applied, in the early stages of treatment. Moreover, in clinical settings, especially in cardiovascular medicine, testing of blood lipid profiles is routine, which paves the way for practical application of these atherosclerosis indexes. In addition, our analysis used data from the CHARLS, which is a large sample-size study of the middle-aged and elderly population in China. Thus, it had good representativeness and high statistical power to discover potential associations. However, our use of data from a single age group in a single country is also a limitation of our study, and thus, caution should be exercised when extending our findings to young people or populations in other countries. Finally, although we found variations in associations in subgroup analyses, some subgroups had smaller sample sizes than others, and thus, further research is needed to validate our findings.

In summary, this study found that high AIPs and high CRI-I and CRI-II values were associated with a high risk of stroke in a population from the CHARLS. Therefore, in view of the increasing burden of lipid-related diseases in China, active interventions in populations with dyslipidemia and monitoring of their atherosclerosis indexes may help to reduce their risk of stroke.
